# Early Histiocytic Pleuritis Revealing Tuberculosis on Repeat Thoracoscopy: A Case Report

**DOI:** 10.7759/cureus.98118

**Published:** 2025-11-29

**Authors:** Usamah Al-Anbagi, Sheikh Jamal, Abdulrahman S Al-Janahi, Muna A Abuhejleh, Abdulqadir J Nashwan, Hatem M Abusriwil

**Affiliations:** 1 Internal Medicine, Hamad Medical Corporation, Doha, QAT; 2 Pulmonology, Hamad Medical Corporation, Doha, QAT; 3 Laboratory Medicine and Pathology, Hamad Medical Corporation, Doha, QAT; 4 Nursing and Midwifery Research, Hamad Medical Corporation, Doha, QAT

**Keywords:** case report, extrapulmonary tuberculosis, histiocytic inflammation, medical thoracoscopy, mycobacterium tuberculosis, pericardial effusion, pleural effusion

## Abstract

Tuberculosis (TB) continues to pose a global health challenge, particularly in its extrapulmonary forms such as pleural and pericardial involvement. These presentations often mimic other conditions and yield inconclusive results on initial microbiological and histopathological testing due to their paucibacillary nature. We report the case of a 45-year-old previously healthy man who presented with recurrent pleural and pericardial effusions. Extensive investigations during the first admission, including pleural fluid analysis, polymerase chain reaction (PCR), and thoracoscopic biopsy, were non-diagnostic. Histopathology initially revealed chronic histiocytic inflammation without necrosis, a finding frequently considered nonspecific but which may represent an early stage of tuberculous pleuritis. The patient improved transiently but presented two months later with contralateral pleural effusion. Repeat thoracoscopy demonstrated multiple “sago-like” nodules, and pleural biopsy confirmed granulomatous inflammation with acid-fast bacilli (AFB). *Mycobacterium tuberculosis* was subsequently cultured, confirming the diagnosis. Anti-TB therapy was initiated with rapid clinical and radiologic improvement. This case highlights that isolated histiocytic inflammation on pleural biopsy should raise suspicion for early tuberculous pleuritis, particularly in endemic regions or in patients with recurrent or unexplained serous effusions. Repeated or targeted thoracoscopic sampling remains crucial when initial results are inconclusive. Early recognition and timely initiation of therapy are essential to prevent complications and improve outcomes in pleuro-pericardial TB.

## Introduction

Tuberculosis (TB) remains one of the most significant infectious diseases worldwide and continues to represent a major global health challenge, with extrapulmonary forms accounting for nearly one-quarter of all reported cases [1]. Among these, pleural and pericardial TB are well-recognized manifestations of extrapulmonary TB that often present with nonspecific clinical features such as fever, chest pain, and dyspnea, frequently occurring in the absence of classical pulmonary findings [2].

Diagnosis in such cases is particularly challenging because these forms are typically paucibacillary, leading to low detection rates with conventional microbiological methods, including smear microscopy, culture, and nucleic acid amplification tests (NAATs) [3]. Furthermore, the histopathological spectrum of tuberculous serositis evolves over time. In early stages, pleural or pericardial involvement may display histiocytic or non-granulomatous inflammation, which is often misinterpreted as nonspecific [4]. With disease progression, classical caseating granulomatous inflammation with identifiable acid-fast bacilli (AFB) becomes evident.

Given these diagnostic challenges, clinicians must maintain a high index of suspicion and consider repeated or targeted sampling, ideally via thoracoscopic or image-guided biopsy, to improve diagnostic accuracy. Thoracoscopy, in particular, allows direct visualization of pleural abnormalities and facilitates targeted biopsy, significantly enhancing diagnostic yield in suspected pleural TB [1-3]. Recognizing this evolving histopathologic continuum, from early histiocytic inflammation to fully developed caseating granulomas, is essential to prevent diagnostic delay.

We present a case of relapsing pleuro-pericardial TB in which initial investigations, including pleural biopsy, were non-diagnostic and revealed only histiocytic inflammation without necrosis. This case highlights the importance of interpreting early histiocytic changes as a potential precursor to TB, integrating clinical, radiological, and pathological findings, and pursuing repeat evaluation when the initial results are inconclusive.

## Case presentation

We present the case of a previously healthy 45-year-old man who underwent a prolonged and challenging diagnostic course for extrapulmonary TB involving both the pleura and pericardium. His clinical course was characterized by relapsing serous effusions, initially inconclusive investigations, and a definitive diagnosis achieved only after repeated, targeted invasive testing.

The patient first presented with a two-week history of fever, cough, and exertional shortness of breath associated with right-sided pleuritic chest pain. He denied weight loss, night sweats, hemoptysis, abdominal pain, vomiting, diarrhea, urinary symptoms, skin rashes, joint pain, headaches, visual disturbances, or focal neurological deficits. There was no history of prior respiratory illness, chronic lung disease, diabetes mellitus, immunosuppressive disorders, or corticosteroid use. He was a non-smoker, abstained from alcohol, and denied extramarital sexual activity. Notably, he reported recent international travel approximately six weeks before the presentation and contact with a sick individual.

On examination, he was conscious, alert, and oriented. Vital signs were stable: temperature 37°C, heart rate 88 bpm, respiratory rate 21 breaths/min, blood pressure 133/67 mmHg, and oxygen saturation 99% on room air. Respiratory examination revealed reduced breath sounds over the right hemithorax; cardiovascular assessment noted muffled S1 and S2 with no murmurs or rubs. The abdomen was soft and non-tender, with no cervical or axillary lymphadenopathy, and neurological and musculoskeletal examinations were unremarkable. Initial laboratory investigations showed normal hematologic, renal, and hepatic function, with an elevated C-reactive protein (Table 1).

**Table 1:** Laboratory investigations. CRP: C-reactive protein; ALT: alanine aminotransferase; AST: aspartate aminotransferase; ALP: alkaline phosphatase; PT: prothrombin time; INR: international normalized ratio; APTT: activated partial thromboplastin time

Parameters	First admission		2nd discharge		Reference values
	On admission	On discharge	On admission	On discharge	
Total leukocytes	6.9	5.5	6.9	7.9	(6.2 x10^3/uL)
Hematocrit	37.3	35.4	42.1	43.3	(40-50%)
Hemoglobin (gm/dL)	12.8	12.3	14	14	(13-17 gm/dL)
Platelet (x10^3/uL)	280	406	202	340	(150-410 x10^3/uL)
CRP mg/L	109	81	22.3	147	(0-5 mg/L)
Serum urea (mmol/L)	2.9	6.4	4.1	5.9	(2.5-7.8)
Serum creatinine (umol/L)	81	77	76	81	(62-106)
Serum potassium K (mmol/L)	3.5	4.3	3.9	4.8	(3.5-5.3)
Serum sodium (mmol/L)	135	137	139	138	(133-146)
Serum calcium (mmol/L)	2.2	2.41	2.27	-	(2.2-2.6)
Serum total protein (gm/L)	77	68	71	76	(60-80)
Serum albumin (gm/L)	35	28	33	28	(35-50)
ALT (IU/L)	40	54	12	23	(0-41)
AST (IU/L)	36	32	16	21	(0-41)
Alkaline phosphatase (U/L)	109	89	73	108	(40–129)
Serum total bilirubin (mg/dl)	13.1	6.4	6	8	(0-21)
PT (seconds)	19	12.3	14	14.3	(9.4-12.5 seconds)
INR	1.6	1.1	1.2	1.3	<1
APTT (seconds)	37.9	36.4	35.3	33.7	(25.1-36.5 seconds)

**Figure 1:**
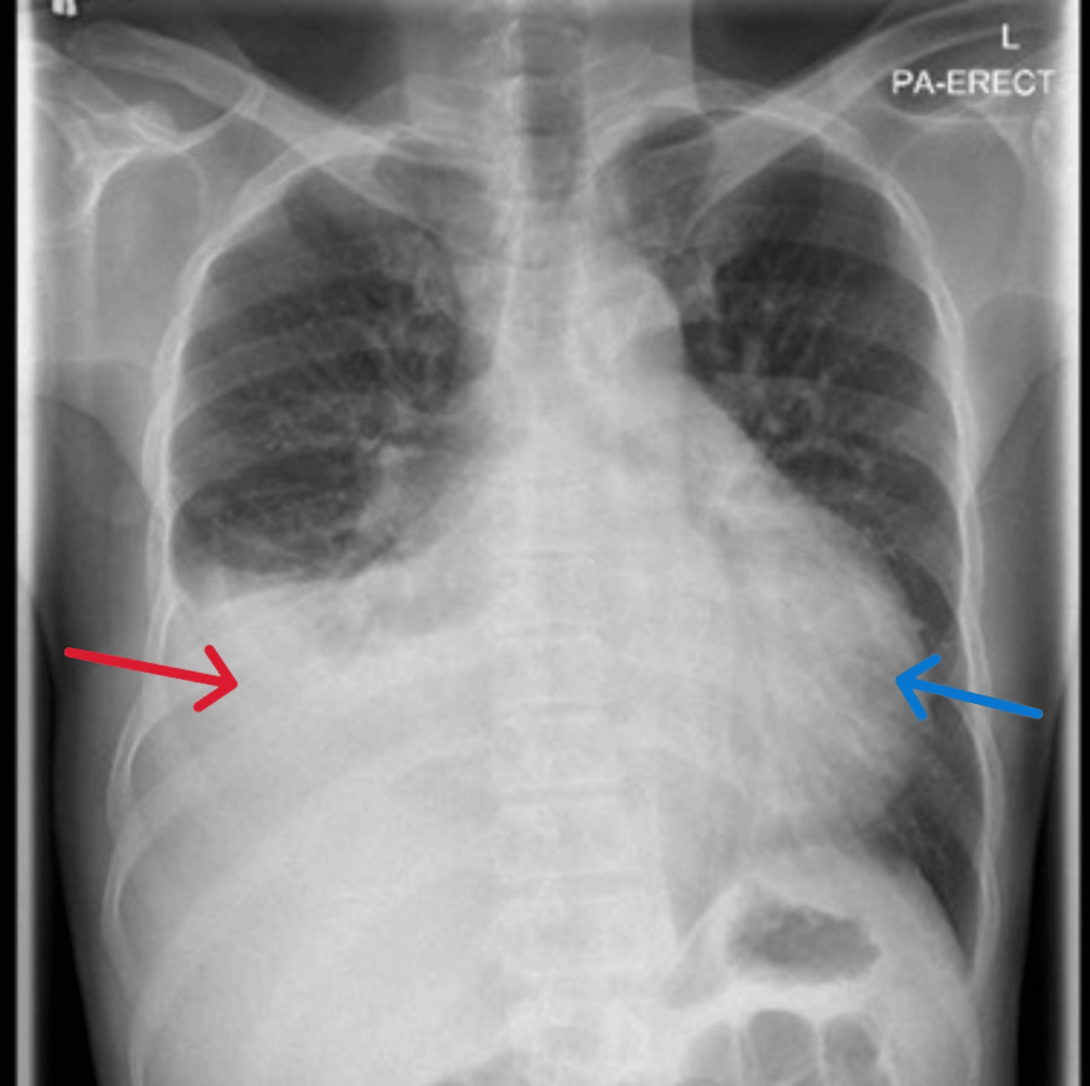
Chest X-ray showing moderate right pleural effusion (red arrow) and cardiomegaly (blue arrow).

Chest radiography revealed cardiomegaly with a right-sided pleural effusion (Figure 1). A working diagnosis of pulmonary or pleural TB was considered, and two sputum samples were sent for AFB smear, polymerase chain reaction (PCR), and culture. Bedside echocardiography demonstrated a large pericardial effusion suggestive of pericardial involvement. He was started empirically on azithromycin (500 mg daily) and ceftriaxone (2 g daily). A comprehensive septic work-up was performed, but sputum AFB smear and PCR were negative for *Mycobacterium tuberculosis*.

**Figure 2:**
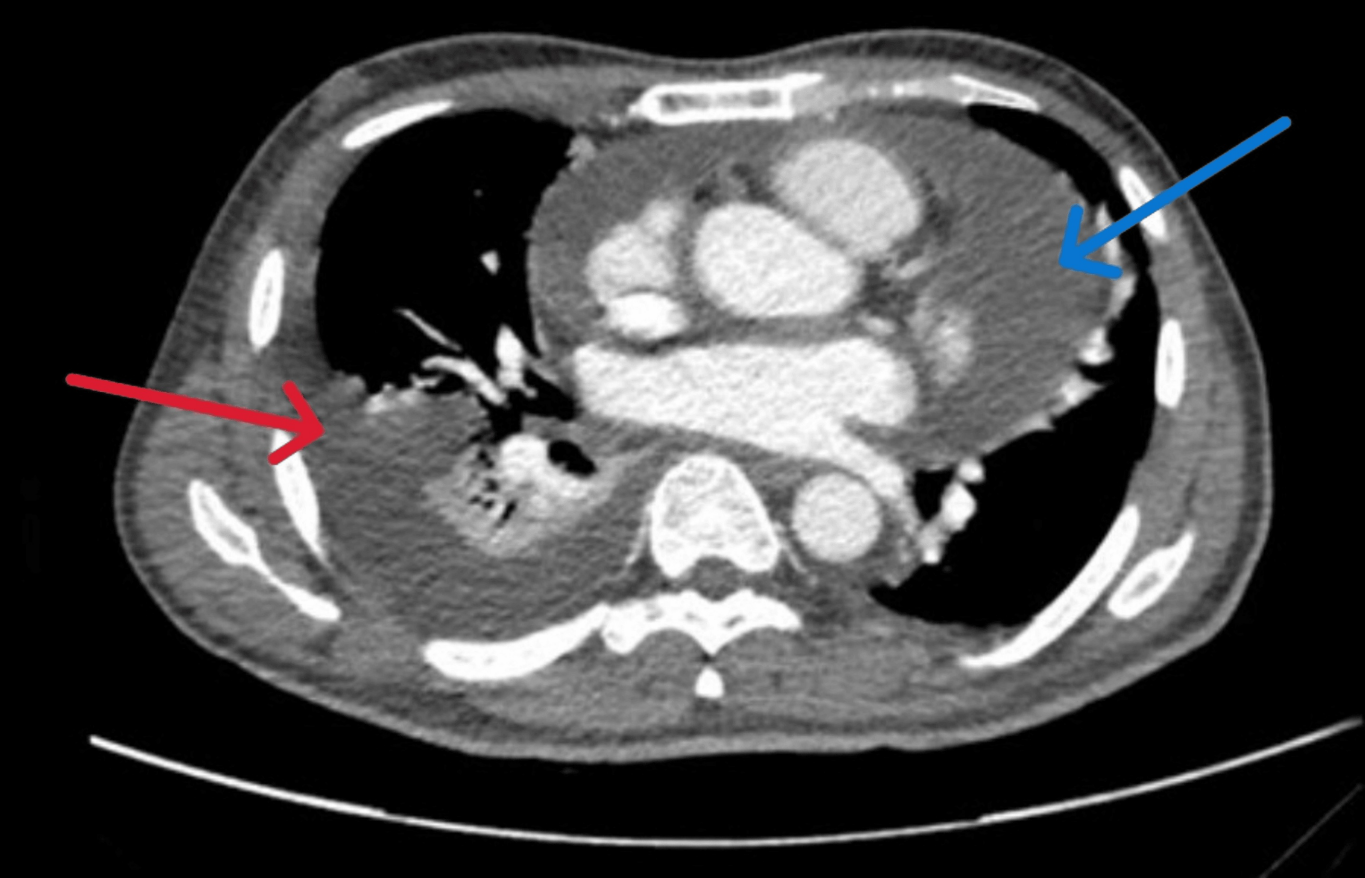
CT chest (axial view) showing right-sided pleural effusion (red arrow) and pericardial effusion (blue arrow).

Persistent pleural effusion prompted diagnostic thoracentesis, which yielded blood-stained exudative fluid (protein 31 g/L; lactate dehydrogenase (LDH) 149 U/L; serum LDH 238 U/L). Cytology was negative for malignancy, and pleural fluid AFB smear, TB PCR, and Gram stain were all negative. Repeat echocardiography three days later showed a moderate pericardial effusion without tamponade. QuantiFERON-TB Gold (QIAGEN N.V., Netherlands), HIV serology, and antinuclear antibody (ANA) profile were negative. Chest CT demonstrated moderate right-sided pleural effusion, left basal consolidation with atelectasis, a small left pleural effusion, and a moderate-to-large pericardial effusion (Figure 2).

**Figure 3:**
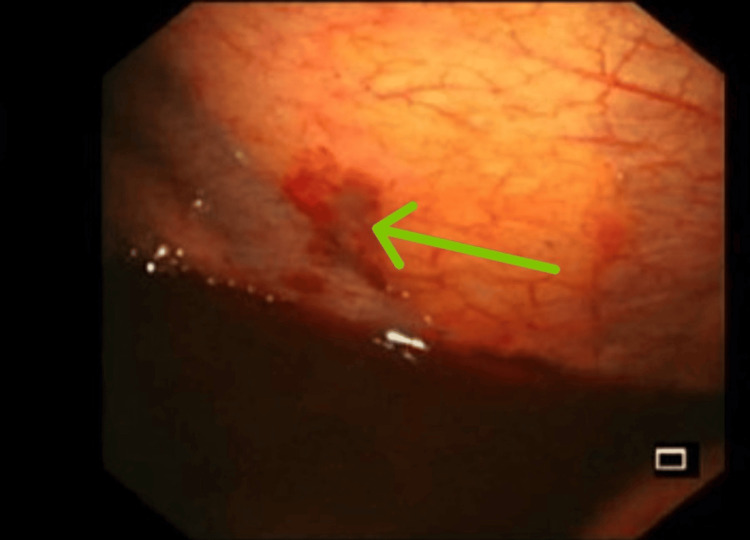
Thoracoscopy showing small dark patches on the parietal pleura and lung surface (green arrow).

Medical thoracoscopy revealed several small dark patches over the parietal pleura and lung surface without adhesions or visible granulomas (Figure 3). Multiple pleural biopsies were taken for histopathology, AFB smear, and TB PCR, all of which were negative.

The patient’s symptoms improved, and he was discharged with instructions for close follow-up while awaiting final results. However, he did not attend his scheduled appointment. Histopathology showed chronic histiocytic inflammation without granulomas or malignancy, and subsequent *Mycobacterium tuberculosis* cultures from sputum, pleural fluid, and pleural biopsy remained negative.

**Figure 4:**
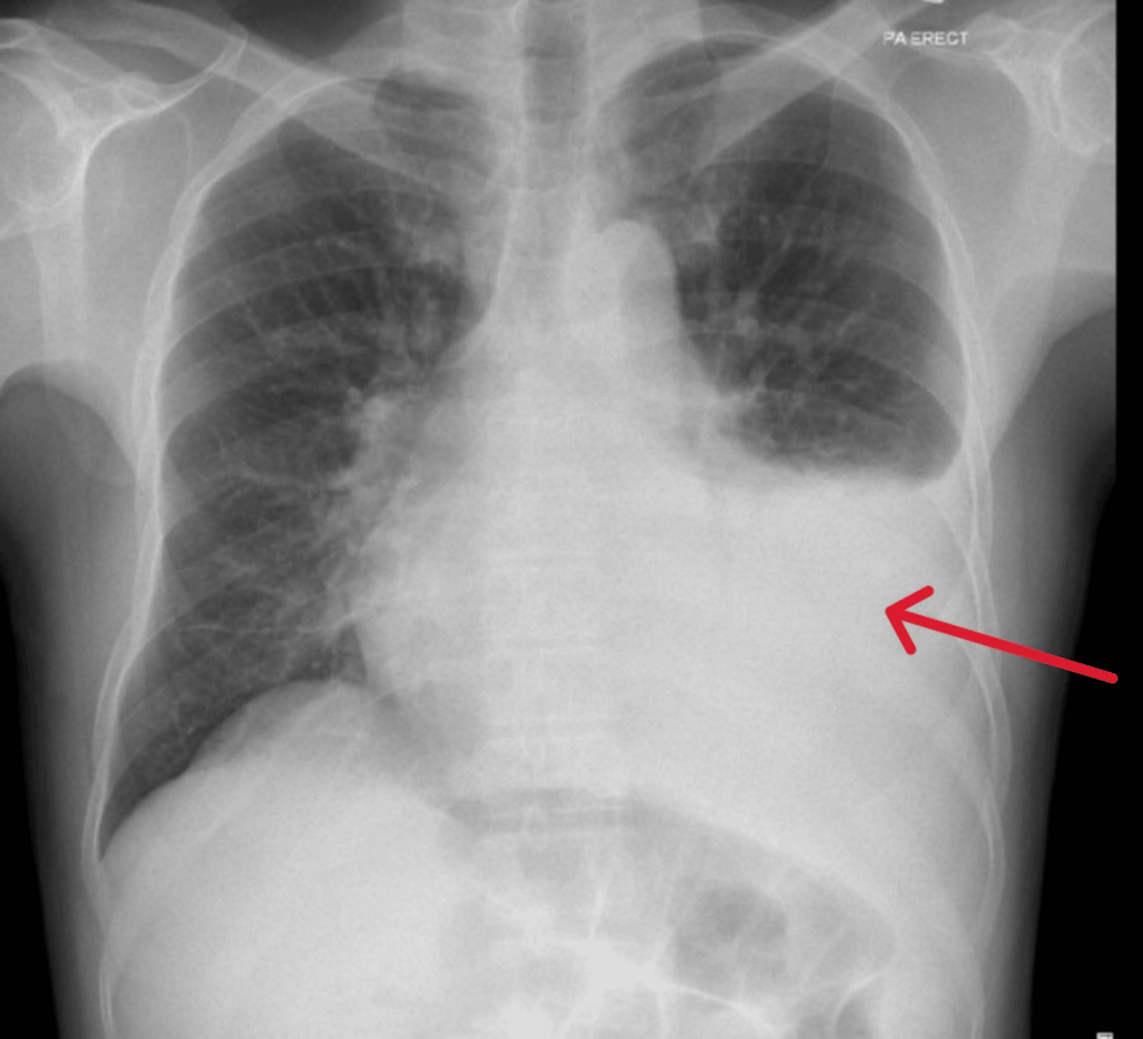
Chest X-ray (posteroanterior view) showing left-sided pleural effusion (red arrow).

Approximately two and a half months later, he re-presented with a three-day history of cough, fever, and mild shortness of breath. He denied chest pain, night sweats, or weight loss and reported no recent travel or sick contacts. Laboratory tests again showed normal organ function with mildly elevated C-reactive protein. Chest radiography now demonstrated a left-sided pleural effusion (Figure 4). Given the prior history, TB was strongly suspected, though sputum AFB smear and TB PCR remained negative.

**Figure 5:**
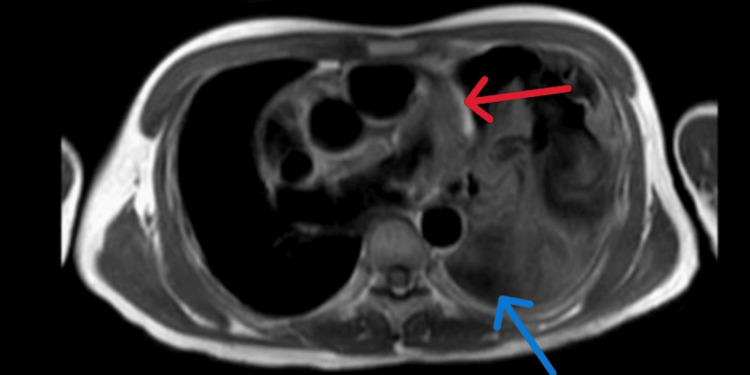
Cardiac MRI (axial view) demonstrating a thickened, enhancing pericardium (red arrow) without pericardial effusion, and a left-sided pleural effusion (blue arrow).

Diagnostic thoracentesis revealed turbid, exudative, lymphocyte-predominant fluid (4,375 cells/µL) with elevated protein and LDH. Cytology was negative for malignant cells, and the AFB smear and TB PCR from the pleural fluid were negative. Echocardiography showed a mild pericardial effusion. CT imaging confirmed a large left pleural effusion with secondary atelectasis and a small pericardial effusion. Cardiac MRI excluded myocarditis but revealed pericardial thickening and enhancement consistent with pericarditis (Figure 5).

**Figure 6:**
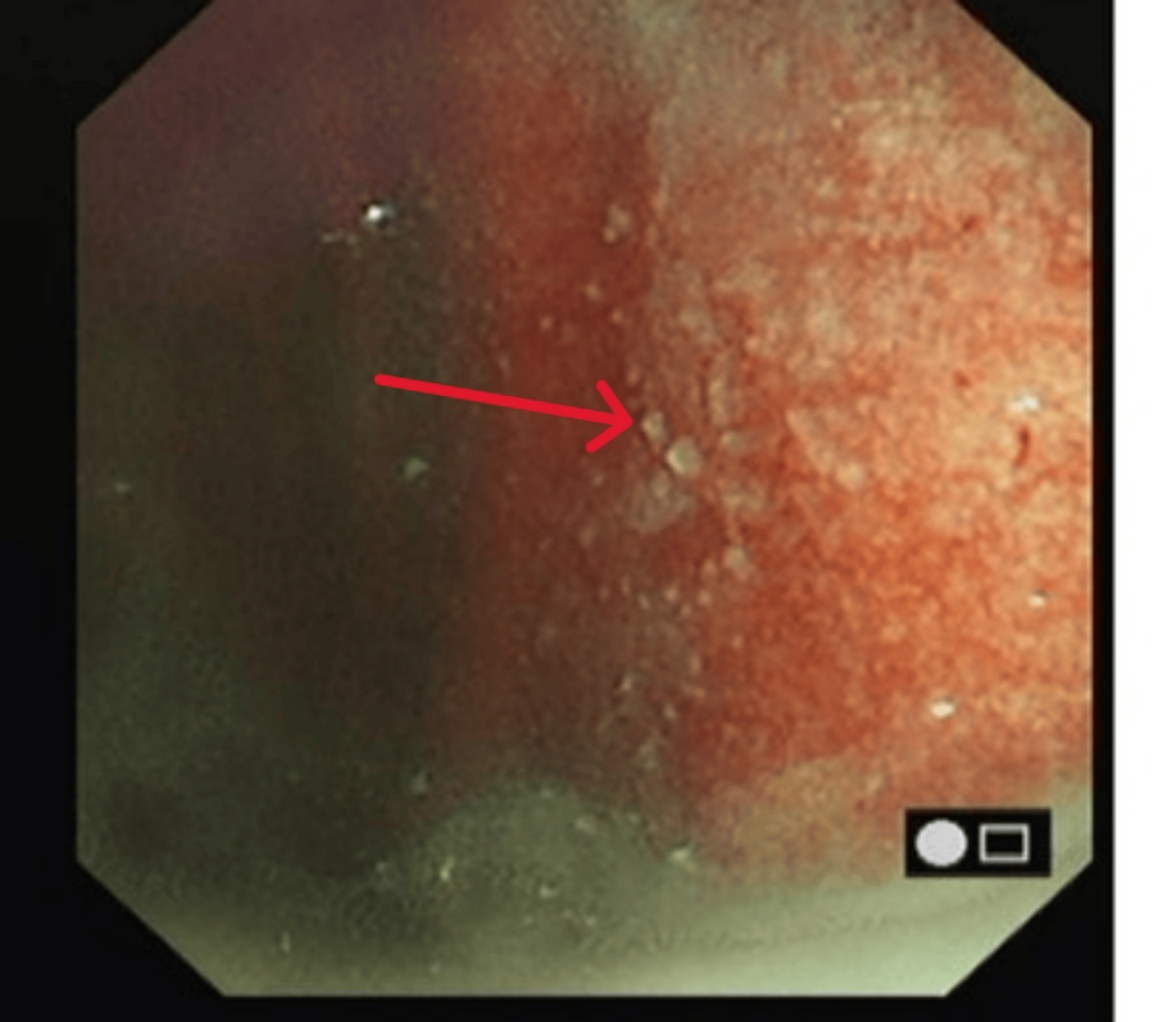
Thoracoscopy image showing classical “sago-like” nodules on the parietal pleura (red arrow).

Following multidisciplinary discussion, a repeat medical thoracoscopy was performed. The parietal pleura appeared studded with classic “sago-like” nodules (Figure 6).

**Figure 7:**
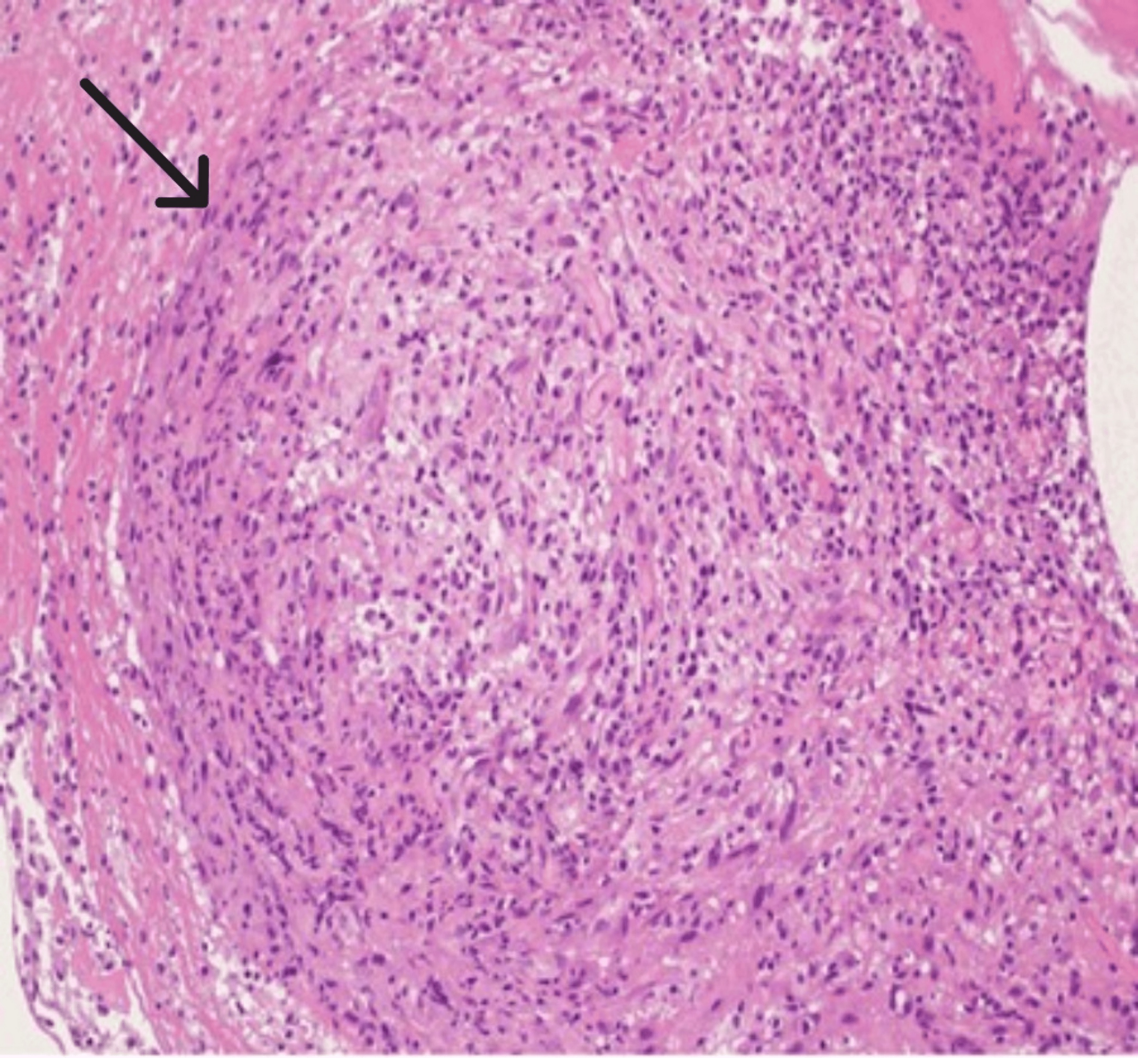
Histological section showing a non-necrotizing granuloma (black arrow) (H&E, ×200 magnification).

**Figure 8:**
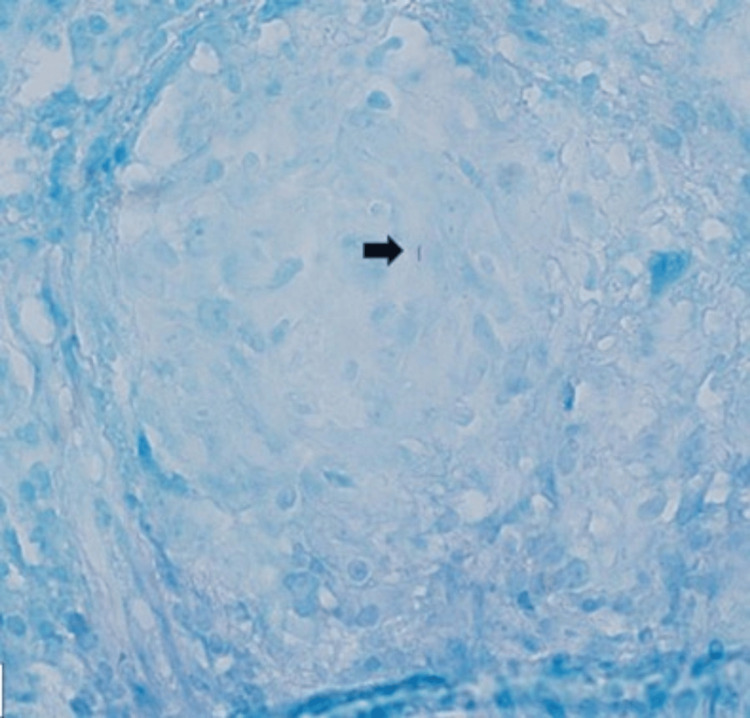
Ziehl-Neelsen stain highlighting bright pink bacillus Mycobacterium (black arrow) (X400).

Multiple biopsies were obtained for culture and histopathology. This time, both the AFB smear and the PCR from the pleural biopsy were positive. Histopathology showed non-necrotizing granulomatous inflammation (Figure 7), and Ziehl-Neelsen staining demonstrated bright pink bacilli consistent with *Mycobacterium tuberculosis* (Figure 8). Fungal stains were negative, and the culture subsequently grew *Mycobacterium tuberculosis*.

The patient was started on standard four-drug anti-TB therapy (rifampicin, isoniazid, pyrazinamide, and ethambutol; 150/75/400/275 mg, four tablets daily). His condition improved significantly, and he was discharged after two weeks in good clinical condition. At follow-up visits at one and six weeks, he remained asymptomatic and clinically well.

## Discussion

TB remains a major global health problem and continues to pose substantial diagnostic challenges, particularly in its extrapulmonary forms such as pleural and pericardial disease. These manifestations are typically paucibacillary, often producing nonspecific clinical and laboratory findings that delay diagnosis and treatment. The present case illustrates the diagnostic difficulties in distinguishing pleuropericardial TB and the significance of histiocytic inflammation as a possible early morphologic stage of tuberculous serositis [1,2].

In this patient, the initial pleural biopsy demonstrated chronic histiocytic inflammation without granulomas or necrosis. Although usually considered nonspecific, such findings may represent an early or “pre-granulomatous” phase of tuberculous infection [4,5]. In early disease, the immune response may be insufficient to form the classical granulomatous architecture, leading to misinterpretation as nonspecific inflammation [4,5]. As the host response matures, the process typically evolves from macrophage-rich infiltrates to non-necrotizing granulomas and eventually to caseating granulomas once bacillary load and immune activation increase.

The sequential histopathologic transformation observed in this case, from histiocytic inflammation on the first biopsy to granulomatous inflammation with AFB positivity on the second, clearly demonstrates this immunopathologic continuum. Recognizing this pattern is crucial, as it underscores the importance of maintaining a high index of suspicion when histiocytic inflammation is observed in patients from TB-endemic regions or with recurrent or unexplained pleural effusions.

Pleuro-pericardial TB remains one of the most diagnostically elusive forms of extrapulmonary TB. Despite extensive initial investigations, including smears, cultures, PCR, and histopathology, results are often negative or inconclusive. This highlights the importance of repeated or targeted sampling, as a second thoracoscopic biopsy may be required to reach a definitive diagnosis, as in this case. The value of repeat thoracoscopy is well documented, particularly when classical “sago-grain” nodules [6] are visualized on the pleural surface. Clinically, pleuropulmonary TB can mimic a variety of conditions, including malignancy, connective tissue disease, or fungal infection, given its overlapping symptoms of fever, chest pain, and dyspnea [2,3].

The paucibacillary nature of the disease contributes to the poor sensitivity of microbiological tests: AFB smears from serous effusions are positive in only 0-10% of cases, while cultures, though specific, yield positive results in up to 58% and require weeks to finalize [7]. Molecular diagnostics, such as Xpert MTB/RIF (Cepheid, USA) and other NAATs, have improved accuracy but remain less sensitive in fluid samples than in tissue [8].

Adenosine deaminase (ADA) and interferon-gamma (IFN-γ) levels can aid diagnosis, with ADA levels >35-40 U/L demonstrating high sensitivity, though false positives may occur in empyema or malignancy; IFN-γ offers superior specificity but is less widely available [9]. Ultimately, tissue biopsy remains the cornerstone of diagnosis, with closed-needle or thoracoscopic biopsies identifying granulomatous inflammation in up to 90% of cases of pleural TB [10].

The sequential involvement of both pleural spaces in this patient likely reflects the evolving nature of tuberculous serositis, possibly due to lymphatic spread or immune-mediated mechanisms. The development of contralateral effusion after initial improvement should therefore heighten suspicion for TB rather than exclude it, especially in endemic settings. Pericardial involvement further complicates the clinical picture. Complications such as cardiac tamponade, effusive-constrictive pericarditis, and chronic constriction are well-recognized [11,12]. Advanced imaging, especially echocardiography and cardiac MRI, is essential for early detection and characterization [13]. In this patient, pericardial thickening and enhancement on MRI strongly supported a tuberculous etiology (Table 2).

**Table 2:** Diagnostic performance of tests used in tuberculous serositis. AFB: acid-fast bacilli; PCR: polymerase chain reaction; ADA: adenosine deaminase; IFN-γ: interferon-gamma; TB: tuberculosis

Diagnostic modality	Sensitivity (%)	Specificity (%)	Advantages	Limitations	Supporting reference links
Pleural/Pericardial Fluid AFB Smear	0-10%	High	Fast, cheap	Very insensitive in paucibacillary TB	[6,7]
Fluid Culture	23-58%	High	Definitive if positive	Prolonged wait, needs specialized media	[6,7]
PCR (fluid)	15-60% (some report 43–77%)	High (up to 99%)	Rapidly detect resistance genes	Lower sensitivity in fluid than in the tin issue	[11]
Biopsy (histology/PCR)	10-64 (PCR to 90% tissue)	High	Higher yield; diagnostic lesions	Invasive, may miss focal lesions	[10,14-16]
ADA (>35–40 U/L)	87-90%	58%	Quick, available	False positives in other conditions	[9,17,18]
IFN-γ	85-97%	94-98%	Excellent performance	Expensive, less available	[9,19]

In many TB-endemic regions, the burden of multidrug-resistant TB (MDR-TB), defined as resistance to at least isoniazid and rifampicin, remains a major public health concern, with prevalence estimates ranging from 3-4% among new cases to more than 18-20% among previously treated individuals [14,15,20]. Management of MDR-TB requires individualized, susceptibility-guided regimens, often incorporating Group A agents such as bedaquiline, linezolid, and levofloxacin/moxifloxacin [16]. Current WHO guidance emphasizes the use of rapid molecular diagnostics (e.g., Xpert MTB/RIF, line-probe assays) for early detection, followed by all-oral longer regimens and close monitoring to reduce toxicity and prevent treatment failure [21]. Strengthening programmatic support, including adherence monitoring, patient-centered care models, and pharmacovigilance, is essential to improving outcomes and preventing further drug resistance in high-burden settings.

This case reinforces the importance of diagnostic persistence and clinical vigilance in complex extrapulmonary TB presentations. Recognition of histiocytic inflammation as an early histopathologic phase of tuberculous serositis, together with repeated or targeted thoracoscopic biopsy when initial findings are inconclusive, is essential for timely diagnosis, early initiation of therapy, and improved patient outcomes.

## Conclusions

This case highlights the diagnostic complexity of extrapulmonary TB, particularly when initial microbiological and histopathological findings are inconclusive. Histiocytic inflammation without granulomas may represent an early stage of tuberculous serositis in paucibacillary disease rather than a nonspecific finding. The patient’s evolution from histiocytic inflammation to granulomatous inflammation with AFB on repeat thoracoscopy exemplifies the immunopathologic continuum of TB. Clinicians should maintain a high index of suspicion in endemic regions and pursue repeat or targeted biopsies when results are indeterminate. Early multidisciplinary collaboration is essential for accurate diagnosis, prompt treatment, and prevention of chronic complications such as constrictive pericarditis or fibrothorax.
